# Analysis of the Drag Reduction Performance and Rheological Properties of Drag-Reducing Additives

**DOI:** 10.3390/polym16091247

**Published:** 2024-04-29

**Authors:** Ailian Chang, Le Huang, Song Wei, Minglu Shao

**Affiliations:** 1 School of Mechanical Engineering and Rail Transit, Changzhou University, Changzhou 213164, China; cal@cczu.edu.cn (A.C.);; 2 Jiangsu Province Engineering Research Center of High-Level Energy and Power Equipment, Changzhou University, Changzhou 213164, China; 3 Collaborative Innovation Center for Water Pollution Control and Water Safety in Karst Area, Guilin University of Technology, Guilin 541004, China; 4 School of Petroleum and Natural Gas Engineering, School of Energy, Changzhou University, Changzhou 213164, China

**Keywords:** polymers, surfactants, rheology, drag reduction, shear-thinning fluid

## Abstract

In the practical application of hydraulic rotating machinery, it is essential to thoroughly explore drag reduction and rheological characteristics of drag-reducing additives to optimize machinery efficiency and reduce equipment consumption. This paper combines simulation and experimental approaches to investigate the drag-reduction performance and rheological properties of drag-reducing additives. Numerical simulations are initially conducted to investigate the shear-thinning properties of drag-reducing fluid and explore variations in drag-reduction rate. Turbulent phenomena characteristics are described by analyzing turbulent statistical quantities. Subsequently, the rheological behaviors of polyethylene oxide (PEO), cetyltrimethyl ammonium chloride (CTAC), and their mixed solutions under different conditions are scrutinized using a rotational rheometer. The findings indicate that the drag reduction effect amplifies as the rheological index *n* and characteristic time *λ* decrease. The numerical simulations show a maximum drag reduction rate of 20.18%. In rheological experiments, a three-stage viscosity variation is observed in single drag-reducing additives: shear thickening, shear thinning, and eventual stabilization. Composite drag-reducing additives significantly reduce the apparent viscosity at low shear rates, thereby strengthening the shear resistance of the system.

## 1. Introduction

Enhancing energy efficiency has become a focal point across various industries due to the continuous growth in energy consumption. Drag reduction technology is widely used in industrial processes such as pipeline crude oil transportation, centralized heating, refrigeration systems, and oil well fracturing, as it offers improved energy efficiency and reduced equipment wear [1,2,3,4]. In industrial production, drag-reducing additives such as high-molecular-weight polymers and surfactants are commonly used. These additives typically exhibit shear-thinning behavior. Therefore, researching their drag reduction and rheological properties is of paramount importance. Currently, scholars, both domestically and internationally, primarily study the laws and mechanisms of drag reduction technology through experiments and numerical simulations [5].

Toms et al. [6] found that introducing trace-soluble additives in turbulent flow can significantly reduce pumping power losses, laying the foundation for drag reduction technology research. Subsequently, scholars have conducted numerous drag reduction and rheological experiments focusing on individual drag-reducing agents. Hong et al. [7] investigated the drag reduction efficiency of two polymer materials using a rotating disk apparatus (RDA). The research showed that in turbulent flow, the efficiency of drag reduction increased with the concentration of polymers. However, beyond a certain concentration, the efficiency of drag reduction decreased. Zhang et al. [8] conducted drag reduction experiments on PEO solutions and achieved a maximum drag reduction rate (DR%) of 18%. The results indicate that the DR% of drag-reducing additives is influenced by time, temperature, velocity, concentration, and molecular weight. Sandoval et al. [9] investigated the drag reduction performance of PEO, Polyacrylamide (PAM), and Xanthan gum (XG) using a pipeline apparatus. They found significant synergistic effects between the polymers. Quan et al. [10] investigated the drag reduction performance of three different polymer materials using a flow loop apparatus. They observed that shear had a significant impact on the drag reduction rate. With an increase in shear time, the drag reduction rate sharply decreased.

Subsequent research has extended the scope of drag reduction technology by investigating composite drag-reducing additives and micro-groove structures. Abdulbari et al. [11] found that the studied grooves, when used in conjunction with trace additives, can achieve high levels of passive drag reduction performance using RDA. Wen et al. [12] conducted experiments and numerical simulations to analyze the flow characteristics and drag reduction mechanisms of micro-grooved rotating discs at different speeds. The results indicated that micro-grooved disks exhibit significant drag reduction effects. Bari et al. [13] investigated the complex of anionic polymers and non-ionic surfactants and demonstrated their capability to enhance drag reduction and mechanistic degradation performance.

Although scholars have researched drag-reducing additives, explanations for related phenomena remain unclear due to the complexity of drag reduction impact conditions. Additionally, the drag reduction mechanism of additives is still not fully understood. To further investigate the drag reduction and rheological properties of shear-thinning fluids, this study employs a combined approach of numerical simulation and rheological experiments. This study analyzed the turbulent statistics and drag reduction rates of shear-thinning fluids under different rotational speed conditions through numerical simulations. Rheological experiments were also conducted to investigate the rheological behavior of the PEO and CTAC mixed systems. The aim of this study is to clarify the viscosity properties, shear-thinning or thickening phenomena, and potential interactions within the mixed system. This research systematically reveals the drag reduction characteristics of surfactants in shear-thinning fluids through the integration of numerical simulation and experimentation. The findings provide a foundational basis for future, in-depth studies.

## 2. Numerical Simulation

### 2.1. Geometry and Mesh

**Figure 1:**
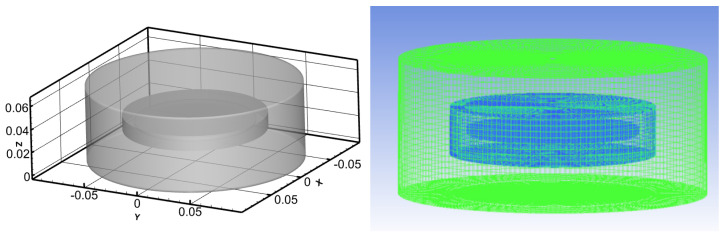
Geometry and mesh.

The RDA consists of a smooth rotating disk and a liquid-containing trough. The cylindrical enclosed liquid-containing trough has a height of H = 65 mm and a radius of R = 90 mm. The rotating disk has a thickness of h = 3 mm and a radius of r = 50 mm. The normal gap between the top of the disk and the top of the liquid-containing disk is S = 32 mm. The numerical simulation employs a transient turbulent flow model and utilizes a non-uniform structured grid division. The geometry and mesh are shown in Figure 1.

### 2.2. Governing Equations and Boundary Conditions

The current computation employs an incompressible fluid, assuming that variations in fluid density are solely induced by changes in temperature while neglecting the effects of viscous dissipation. The governing equations for the fluid consist of the continuity equation and the momentum equation, as depicted in Equations (1) and (2), respectively [14].

The continuity equation:

(1) $$\frac{\partial \bar{u_{i}}}{\partial x_{i}}=0$$

The momentum equation:

(2) $$\frac{\partial \bar{u_{i}}}{\partial t}+\frac{\partial (\bar{u_{i}}\bar{u_{j}})}{\partial x_{j}}=-\frac{1}{\rho }\frac{\partial \bar{p}}{\partial x_{i}}+\frac{\partial }{\partial x_{i}}(\nu \frac{\partial \bar{u_{i}}}{\partial x_{i}}-\bar{u_{i}^{\prime }u_{j}^{\prime }})$$

where *t* is time (s), *p* is pressure (Pa), *u* is the velocity vector (m/s), and *ρ* is the density of the fluid (kg/m^3^). In this study, the Reynolds stress model is employed, which is suitable for strong swirling flow fields (such as curved pipes, rotation, cyclone separators, etc.) [15]. The Reynolds stress equation is depicted as follows in Equation (3):

(3) $$\tau_{ij}^{\prime }=-\rho \bar{u_{i}^{\prime }u_{j}^{\prime }}$$

The selected turbulent model is the Reynolds stress model. The transport equation is shown in Equation (4).

(4) $$\frac{\partial }{\partial t}\bar{u_{i}^{\prime }u_{j}^{\prime }}+\bar{u_{k}}\frac{\partial }{\partial x_{k}}\bar{u_{i}^{\prime }u_{j}^{\prime }}=D_{ij}+\varphi_{ij}-\varepsilon_{ij}+P_{ij}$$

*D_ij_* represents the turbulent kinetic energy diffusion term; *φ_ij_* denotes the pressure–strain term; *ε_ij_* signifies the dissipation term; *P_ij_* stands for the stress generation term.

The constitutive equation model of generalized Newtonian fluid (Carreau–Bird model) is employed, as shown in Equation (5). The Carreau model accurately describes power law behavior at low and high shear rates. Modifying the characteristic time and rheological index in the constitutive equation allows for the simulation and analysis of the shear-thinning characteristics of drag-reducing fluid in drag reduction.

(5) $$\mu_{A}=\mu_{\infty }+(\mu_{0}-\mu_{\infty }){\left[1+{\left(\lambda \dot{\gamma }\right)}^{2}\right]}^{\frac{\left(n-1\right)}{2}}$$

In Equation (5), *n* represents the rheological index, where *n* = 1 corresponds to Newtonian fluid behavior and *n* < 1 signifies shear-thinning fluid behavior. *λ* denotes the characteristic time of the fluid. *μ*_0_ and *μ*_∞_, respectively, represent the apparent viscosity of the liquid phase at zero shear rate and infinite shear rate [16].

The boundary conditions are defined as follows: The interface connects the rotor and stator at the boundary. The Simple algorithm is selected to couple the velocity and pressure fields. The gradient terms are discretized using the least squares method based on the grid center. The momentum equation is discretized using a second-order upwind scheme, while the pressure term is discretized using a second-order interpolation method. A first-order upwind scheme is used to discretize turbulent kinetic energy, turbulent dissipation rate, and Reynolds stress. The calculation employs a hybrid initialization after parameter setup, and convergence is determined when the monitored torque values become steady over time.

### 2.3. Relevance and Accuracy Verification

**Figure 2:**
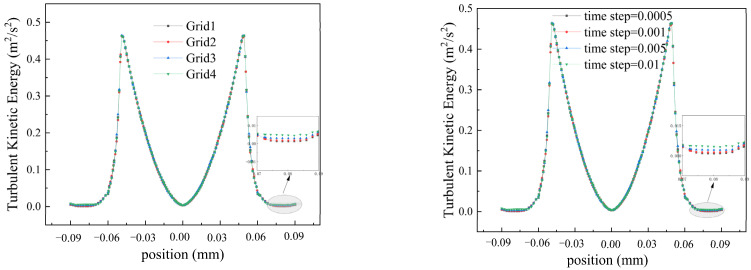
Grids and time steps validation.

To ensure accurate computational results, four grid systems (Grid1, Grid2, Grid3, and Grid4) are established for validation, each with varying numbers of grid nodes. Under the most demanding conditions (rotational speed of 2200), discrepancies are observed between Grid3, Grid4, and Grid1, leading to their exclusion. As the number of grid nodes increased, Grid2 results gradually approached those of Grid1, demonstrating good consistency. Grid2 was chosen for computation due to its reduced computational costs and accurate results. We tested four different time step sizes (0.0005 s, 0.001 s, 0.0015 s, and 0.002 s) to observe trends in turbulent kinetic energy. The results showed that decreasing the time step sizes stabilized the computations. Notably, at 0.0005 s, turbulent kinetic energy closely matched that of 0.001 s. Therefore, we selected a time step size of 0.001 s for computation (see Figure 2).

To verify the accuracy of the results, the calculated torque values were compared with the empirical formula reported in the literature [17], as shown in Equation (6).

(6) M=0.1022asa0.1μ2ρRe1.8

In the equation, a represents the radius of the rotating disc (mm), *s* represents the normal gap between the disc surface and the top of the liquid meniscus in the vessel (mm), and *μ* represents the viscosity (mPa·s).

**Figure 3:**
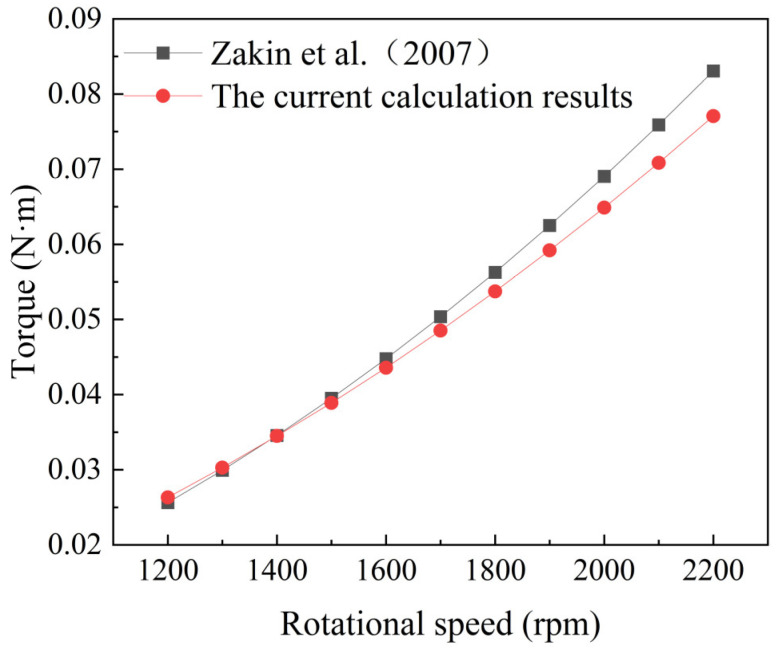
Accuracy validation [17].

Figure 3 illustrates the comparison of the data. The results indicate that the torque values corresponding to the rotational Reynolds number at medium to low rotation speeds are generally consistent with those reported in reference. However, at high speeds, some discrepancies are observed. This is attributed to the phenomenon where excessively high speeds at the disk periphery lead to the formation of jet flows impacting the walls of the trough, causing instability in the vertical boundary layer within the trough and inducing fluctuations throughout the flow domain. Nonetheless, the computed results closely match those obtained from the empirical formula reported in the literature [17], with a maximum error of 7.2% observed at a rotational speed of 2200 rpm. Given the alignment with computational requirements, the numerical simulation method employed in this study is deemed reliable.

### 2.4. Drag Reduction Calculation

**Figure 4:**
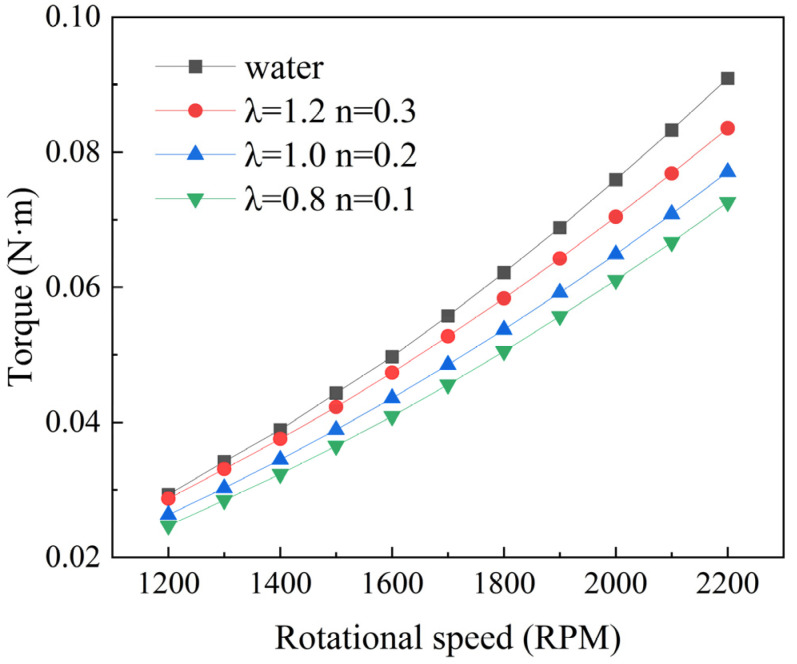
Torque variation trends.

This study utilized the RDA model to calculate torque for water and shear-thinning fluids under turbulent flow conditions at varying speeds. Figure 4 displays the torque values across a range of speeds, from 1200 to 2200 rpm. It is observed that as the speed increases, the torque values also increase, and the torque value for water is greater than that for shear-thinning fluids. The drag reduction rate for the corresponding operating conditions was calculated using Equation (7).

(7) $$DR\%=\frac{T_{w}-T_{s}}{T_{w}}\times 100\%$$

In Equation (7), *T_w_* represents the torque value for the Newtonian fluid, and *T_s_* represents the torque value for the shear-thinning fluid, both measured in units of N·m.

**Figure 5:**
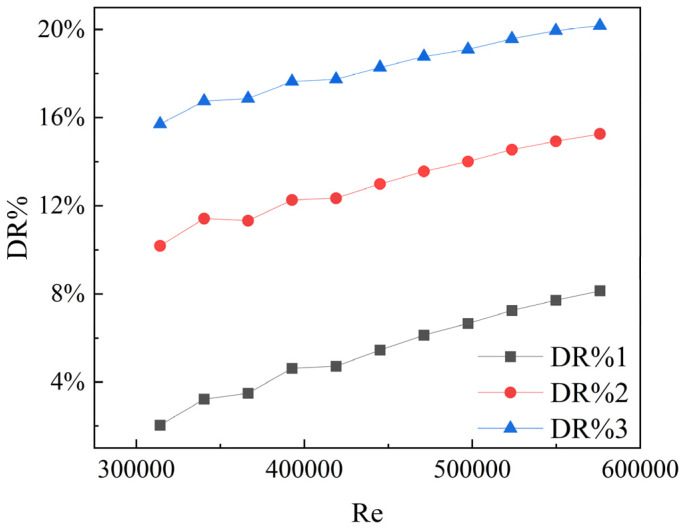
Variation trends of drag reduction.

This study employs water as a reference group for computation and combines the Carreau–Bird model to simulate shear-thinning fluid flow. According to reference [18], the characteristic parameters for three shear-thinning fluids, C1, C2, and C3, are as follows: In the C1 condition, the characteristic time *λ* is 1.2, and the rheological index *n* is 0.3. In the C2 condition, *λ* is 1, and *n* is 0.2. In the C3 condition, *λ* is 0.8, and *n* is 0.1. In this model, the zero-shear viscosity and infinite-shear viscosity are specified as 0.1 and 0.0001, respectively. Based on the computational results, the drag reduction rates for the three shear-thinning fluids are depicted in Figure 5.

### 2.5. Analysis of Turbulence Statistics

#### 2.5.1. Velocity Field

**Figure 6:**
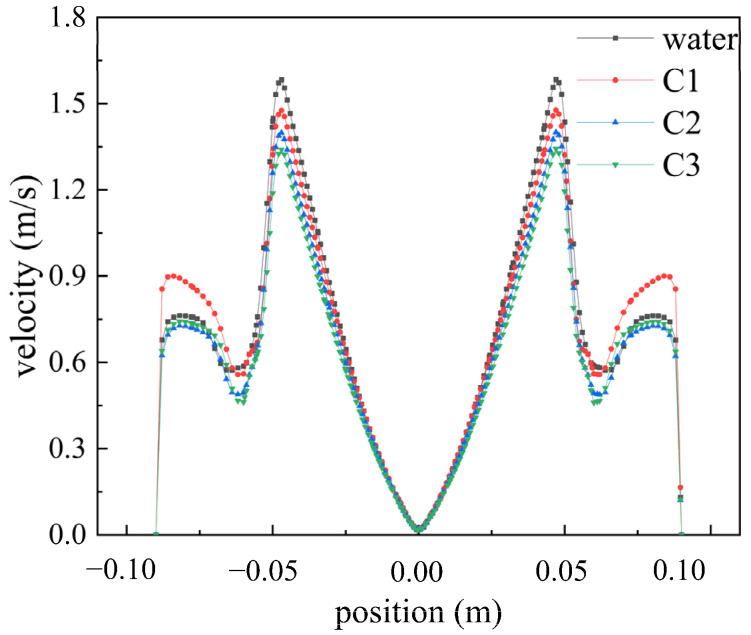
Velocity distribution of different fluids at 2200 rpm.

To investigate the influence of different fluids on the velocity distribution within the flow field, Figure 6 illustrates the velocity distribution of Newtonian fluid (water) and three types of shear-thinning fluids at a speed of 2200 rpm. In both water and shear-thinning fluids, the velocity field exhibits two peaks. In the first stage, from the center to the edge of the disk, the velocity gradually increases to a peak, after which the rotation of the disk drives the fluid domain to rotate. In the second stage, under high-speed rotation, jet flows formed at the edge of the disk accelerate towards the wall, resulting in smaller peaks consistent with the conclusions of reference [19]. Under these conditions, at 2200 rpm, the maximum velocity of water is 1.582 m/s, while the maximum velocities of shear-thinning fluids C1, C2, and C3 are 1.476 m/s, 1.398 m/s, and 1.341 m/s, respectively. As the rheological index *n* and characteristic time *λ* decrease, the peak velocity in the first stage gradually decreases, attributed to the influence of shear characteristics on the velocity distribution, resulting in decreased viscosity of the fluid under high shear forces.

**Figure 7:**
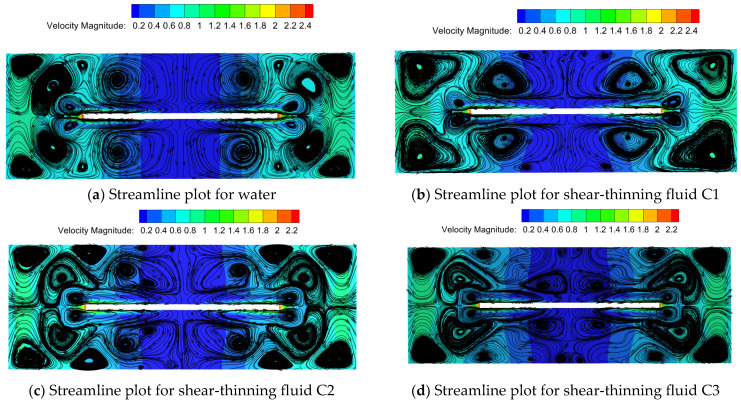
Velocity streamline diagram of several fluids at 2200 rpm.

Figure 7 depicts velocity streamline plots for Newtonian fluid water and three types of shear-thinning fluids at a rotational speed of 2200 rpm. Compared to water, the streamline distribution of shear-thinning fluids appears more chaotic, particularly in the regions directly above and below the disk, where more small vortices form, altering the direction of surface flow. As the characteristic time *λ* and rheological index *n* decrease in shear-thinning fluids, the degree of shear thinning in the fluid above and below the disk becomes more pronounced. Larger vortices transform into smaller ones, generating more small vortices, which reduce fluid flow resistance and achieve drag reduction [20].

#### 2.5.2. Turbulent Dissipation Rate

**Figure 8:**
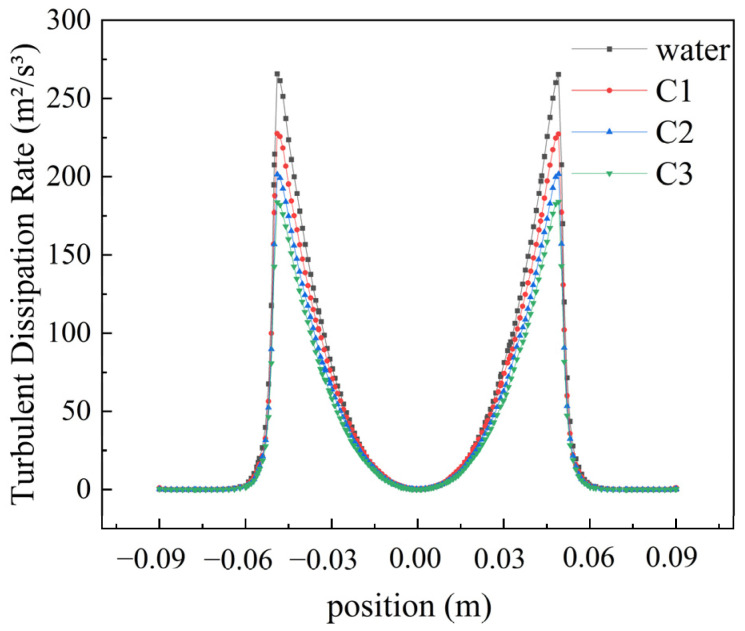
Turbulent dissipation rate distribution of different fluids at 2200 rpm.

Figure 8 illustrates the distribution pattern of turbulent dissipation rates. At a rotational speed of 2200 rpm, the maximum turbulent dissipation rate for water is 265.724 m^2^/s^3^, while for shear-thinning fluids C1, C2, and C3, the maximum values are 227.584 m^2^/s^3^, 201.389 m^2^/s^3^, and 183.686 m^2^/s^3^, respectively. The turbulent dissipation rate gradually increases with the distance from the center of the rotating disk until reaching a peak, then sharply decreases, ultimately approaching zero near the walls of the tank.

#### 2.5.3. Turbulent Kinetic Energy

**Figure 9:**
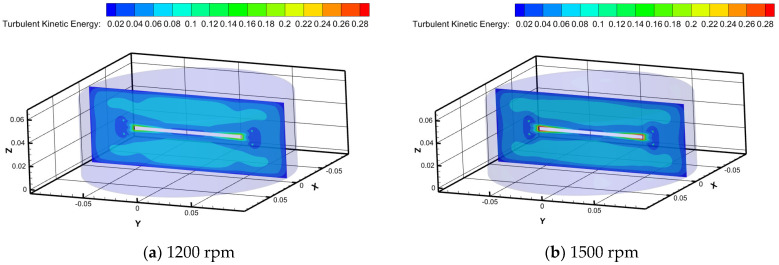

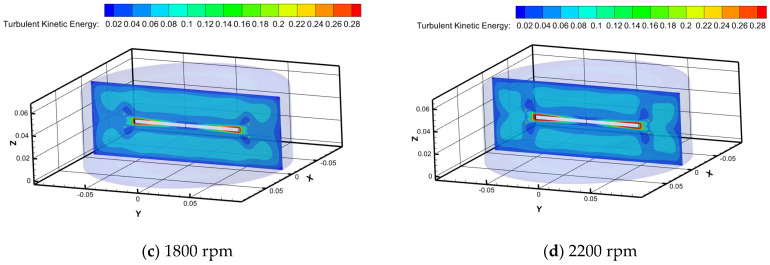
Turbulent kinetic energy clouds at different rotational speeds.

Figure 9 illustrates distinct characteristics of turbulent kinetic energy maps for Newtonian fluid water at different rotational speeds in the RDA. Turbulent kinetic energy reaches its maximum value at the edge of the rotating disk as the rotational speed increases, while it minimizes near the walls of the tank. It is worth noting that the jetting effect at the disk’s edge becomes more pronounced with increasing rotational speed, leading to a corresponding increase in turbulent kinetic energy. At a rotational speed of 1800 rpm, the distribution bands of turbulent kinetic energy begin to break and then recombine, gradually approaching the walls of the tank. This phenomenon is closely related to previous studies on the formation of self-healing micelle structures by surfactants [21].

## 3. Rheological Experiments

### 3.1. Materials

The high-molecular-weight polymer drag-reducing additive utilized in this study is PEO, while the surfactant employed is CTAC, and the organic counterion selected is sodium salicylate (NaSal). The solvent utilized is deionized water. Zhang et al. [22] studied the ratio of cationic surfactant to counterion salt, determining the optimal ratio. When CTAC is dissolved in deionized water with NaSal at a mass ratio of 1:1, the cations on the CTAC molecule are neutralized by the counterion salt. As a result, surfactant molecules in the drag-reducing solution can aggregate to form a more stable micellar drag-reducing structure, achieving the best drag-reduction effect. The specific details of the drag-reducing additives used in the experiment are shown in Table 1.

In addition, rheological tests were conducted to investigate the effects of drag-reducing additive type, temperature, concentration, and shear rate on rheological properties. The concentration conditions studied included three low-concentration solutions: 10 ppm, 20 ppm, and 30 ppm. The temperature conditions were set at 20 °C, 30 °C, and 40 °C. The range of shear rate varied from 0.1 to 1000 s^−1^. Drag-reducing additives were tested separately for single PEO solution, CTAC/NaSal solution, and their composite solution, aiming to derive viscosity (mPa·s) and shear stress (Pa) variation curves with respect to shear rate under corresponding operating conditions.

### 3.2. Experimental Apparatus

**Figure 10:**
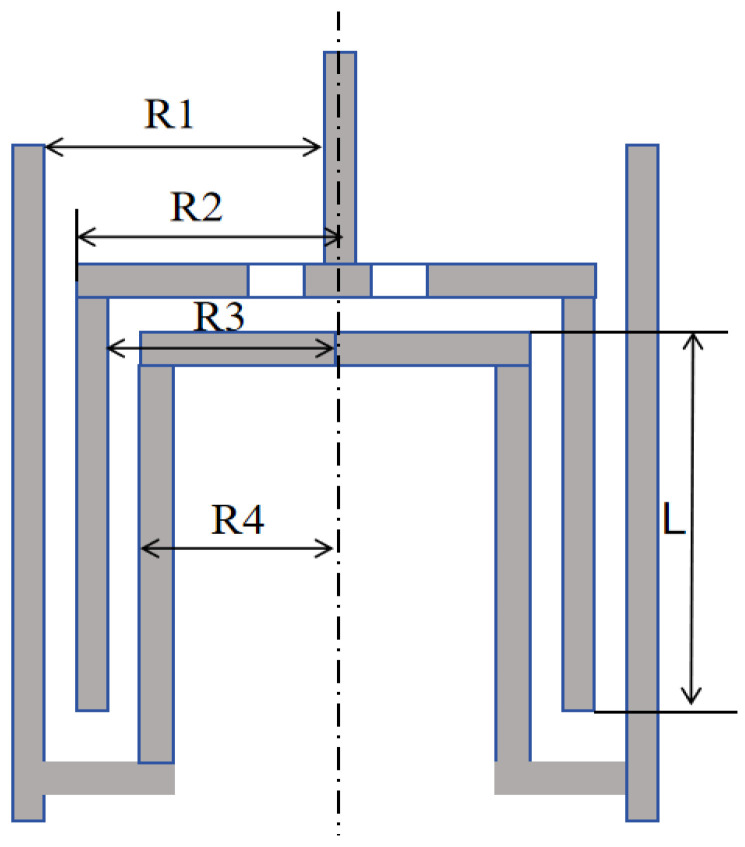
Schematic diagram of double-gap fixture.

The experimental section utilized the MCR 302 rheometer produced by Anton Paar, located in Graz, Austria, to test the rheological properties of drag-reducing additives. Due to the low-viscosity shear-thinning nature of the solution under investigation, a double-gap system fixture was chosen. This fixture is suitable for samples with viscosities below 100 mPa·s, ensuring constant internal temperature and requiring less sample solution due to its small gap. The structural diagram of the fixture is illustrated in Figure 10. The lengths of R1, R2, R3, and R4 are, respectively, 13.798 mm, 13.329 mm, 12.329 mm, 11.912 mm, and 40.000 mm.

### 3.3. Analysis of Rheological Experiments

#### 3.3.1. Rheological Properties of Polymer PEO

**Figure 11:**
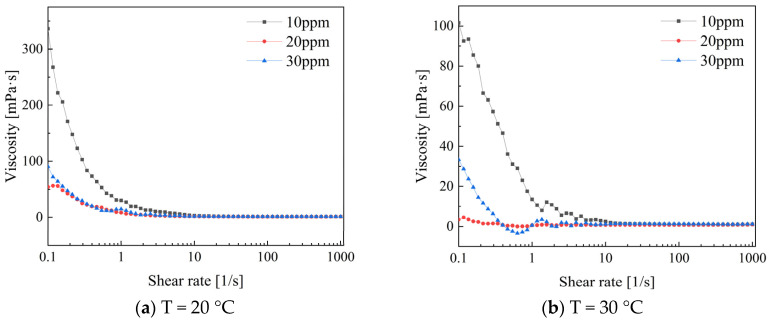
Variation in viscosity of low concentration PEO solution with shear rate.

Figure 11 shows the viscosity variation trend for different concentrations of PEO. The results indicate clear observation of shear-thinning non-Newtonian behavior. The apparent viscosity change in the drag-reducing additives is more pronounced at low shear rates than at high shear rates. At temperatures of 20 °C and 30 °C, the viscosity values show a significant decrease when the shear rate ranges from 0.1 to 10. The apparent viscosity decreases most rapidly at a concentration of 10 ppm for PEO solutions, while the apparent viscosity decrease for concentrations of 20 ppm and 30 ppm is relatively moderate. At low shear rates, the viscosity of shear-thinning fluids changes significantly due to the reversible rearrangement of microstructures within the fluid. This phenomenon can be explained by the relatively low shear forces causing the rearrangement, resulting in an overall increase in viscosity. In contrast, at high shear rates, the fluid’s internal structure is fully disrupted due to strong shear forces. This allows molecules or particles to flow more freely under high-speed shear, resulting in reduced flow resistance within the system. Consequently, this causes relatively smaller changes in viscosity [23].

#### 3.3.2. Rheological Properties of CTAC

**Figure 12:**
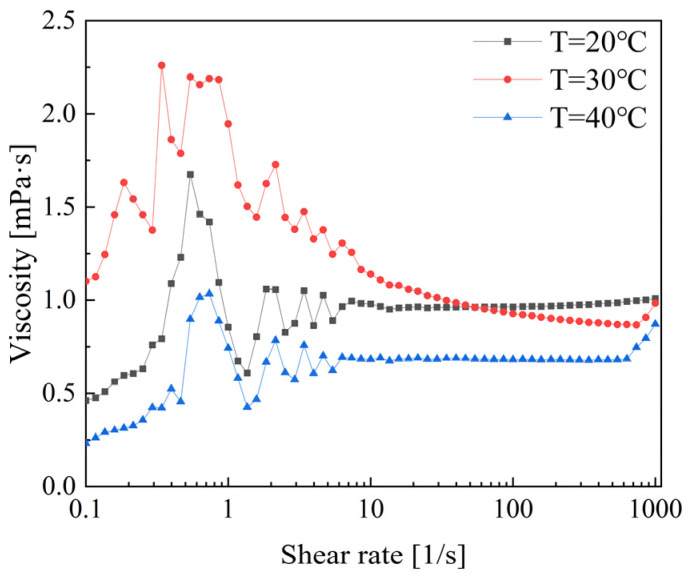
Viscosity variation in CTAC/NaSal solution.

Figure 12 shows the viscosity variation trends of CTAC. The rheological properties can be roughly described in three stages: shear thickening, shear thinning, and finally stabilizing. During the shear thickening stage, the viscosity sharply increases to a peak in the shear rate range of 0.1 to 1. In the shear rate range of 1 to 10, shear thinning occurs, resulting in a decrease in viscosity for all three temperature conditions. The viscosity values begin to stabilize when the shear rate exceeds 10 s^−1^. Notably, at a temperature of 30 °C, the viscosity gradually decreases in the shear rate range of 10 to 1000. However, the viscosity values remain relatively stable in the high shear rate range, with little variation at both 20 °C and 40 °C. Therefore, the temperature of 30 °C demonstrates better performance in terms of drag reduction and rheology [24].

#### 3.3.3. Rheological Properties of Composite Drag-Reducing Additives

**Figure 13:**
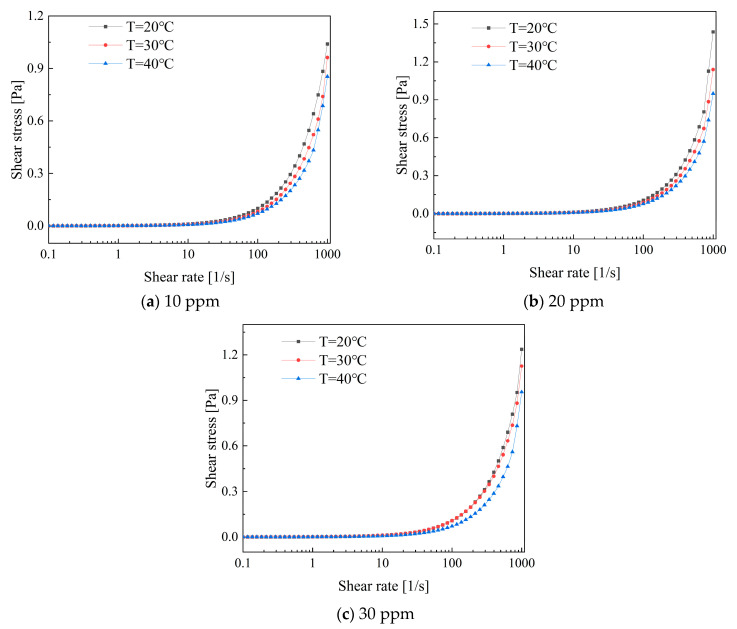
Shear stress variations in PEO and CTAC/NaSal solutions versus shear rates.

Figure 13 illustrates the variation in shear stress with shear rate for a low-concentration composite additive solution. It can be observed that the shear stress increases with an increase in shear rate. The shear stress exhibits a rapid increase within the shear rate range of 100 to 1000. Furthermore, the variation in shear stress is closely linked to temperature. Shear stress is higher at low temperatures than at higher temperatures due to the decrease in apparent viscosity within the solution caused by an increase in temperature. This promotes movement between molecular structures, resulting in a reduction in shear stress.

**Figure 14:**
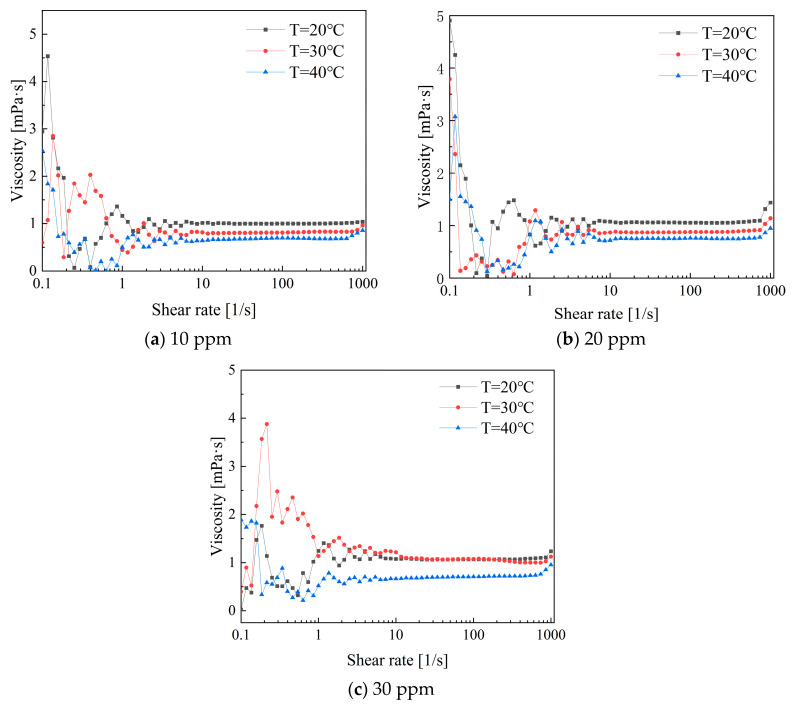
Viscosity variations in PEO and CTAC/NaSal solutions versus shear rates.

Figure 14 illustrates the variation in viscosity with shear rate for low-concentration PEO combined with CTAC/NaSal composite solutions. Compared to single drag-reducing additives like PEO and CTAC, the rheological properties of composite drag-reducing additives are more complex. Within the low shear rate range of 0.1 to 1, the maximum viscosity of the composite solution is lower than that of the single solution. At concentrations of 10 ppm and 20 ppm, the viscosity trend first exhibits shear-thinning, followed by shear thickening, and finally stabilizes after reaching a shear rate of 10 s^−1^. During the stable stage, the viscosity at 20 °C is greater than that at 30 °C, while the viscosity at 40 °C is the lowest [25].

## 4. Results and Discussion

In this study, we initially investigated the drag reduction characteristics of shear-thinning fluids using numerical simulation methods, analyzing turbulence statistics such as velocity, turbulent kinetic energy, and turbulent dissipation rate. Subsequently, experimental analysis was conducted to assess the rheological properties of the polymer PEO, the surfactant CTAC, and their composite solution, elucidating the patterns of variation in shear stress and viscosity with shear rate. The detailed flow field information provided by numerical simulation under various conditions was utilized to analyze the rheological characteristics of additives with shear-thinning properties, thereby summarizing the principles of drag-reducing additives for enhanced application in drag-reduction scenarios. 

In the numerical simulation work, the drag reduction rate of simulated shear-thinning fluids was poor under low shear conditions but improved with increasing Reynolds numbers. This corresponds to the subsequent experimental study on viscosity variation patterns. In the low-shear rate regime, the predominant internal molecular structures of the additives were entangled long-chain structures and single micelles. However, at high shear rates and suitable temperatures, a network structure favorable for drag reduction was formed. During this stage, there was a significant decrease in viscosity. After the shear stress reached 800 s^−1^, the stable network structure could no longer withstand high shear forces, leading to the disruption of the drag-reducing structure into single micelles, resulting in an increase in viscosity.

The rheological experimental results of the composite additive solution indicate that the increase in temperature accelerates the shear transformation of the internal structure of the composite solution, leading to the decomposition of large molecular cluster structures into flexible small-molecule micelles and chain structures under thermal effects, thereby reducing the apparent viscosity of the solution. Additionally, an interesting phenomenon is observed when the shear rate exceeds 970 s^−1^, where a small range of shear thickening occurs, resulting in an increase in viscosity from a stable state. This phenomenon indicates that the composite solution loses its drag-reducing ability as the long-chain structure of PEO can no longer effectively bind with the micelle structure of the CTAC solution, causing the stable spatial network structure to completely rupture. Compared to single-additive solutions, the composite solution significantly reduces the apparent viscosity at low shear rates. Additionally, under stable conditions, the apparent viscosity at high temperatures is lower overall than that at low temperatures. The composite solution demonstrates excellent performance from the initial stages while also incorporating the ability of the CTAC/NaSal solution to withstand high shear forces.

## 5. Conclusions

The main conclusions of this study are listed as follows:

(1) Shear-thinning fluids exhibit excellent drag reduction performance. Among the three types studied, the maximum drag reduction rate reached 20.18%. The drag reduction rate increases with decreasing rheological index *n* and characteristic time *λ* of shear-thinning fluids. This pattern provides valuable insights for selecting drag-reducing additives in industrial production.
(2) The rheological properties of the single drag-reducing additives are influenced by a combination of temperature, concentration, and shear rate. The viscosity changes can be described in three stages: shear thickening, shear thinning, and eventually reaching a steady state.
(3) In the study of solutions involving polymer-surfactant complexes, compared to solutions with single additives, composite solutions notably reduce the apparent viscosity at low shear rates. Additionally, under stable conditions, the overall apparent viscosity at high temperatures is lower than that at low temperatures. These composite solutions exhibit excellent performance from the initial stage and also have the ability to resist high shear when combined with CTAC/NaSal solutions.

## Figures and Tables

**Table 1:** Drag-Reducing Additives.

Additives	Relative Molecular Mass	Purity and Shape	Suppliers
PEO	7 × 10^6^	Powder, 99%	Shanghai Chenqi Chemical Technology Co., Ltd., Shanghai, China
CTAC	320	Powder, 99%	Shanghai Civic Chemical Technology Co., Ltd., Shanghai, China
NaSal	160.1	Powder, 99.5%	Shanghai Maclean Chemical Co., Ltd., Shanghai, China

## Data Availability

All the data in this article were obtained by the author’s field measurements. If necessary, please contact the author by email. The data presented in this study are available on request from the corresponding author.
